# Slc15a4, a Gene Required for pDC Sensing of TLR Ligands, Is Required to Control Persistent Viral Infection

**DOI:** 10.1371/journal.ppat.1002915

**Published:** 2012-09-13

**Authors:** Amanda L. Blasius, Philippe Krebs, Brian M. Sullivan, Michael B. Oldstone, Daniel L. Popkin

**Affiliations:** 1 Departments of Genetics and Immunology, The Scripps Research Institute, La Jolla, California, United States of America; 2 Institute of Pathology, University of Bern, Bern, Switzerland; 3 Department of Immunology and Microbial Science, The Scripps Research Institute, La Jolla, California, United States of America; 4 Departments of Dermatology, Pathology, Molecular Biology and Microbiology, Case Western Reserve University, Cleveland, Ohio, United States of America; University of Pennsylvania, United States of America

## Abstract

Plasmacytoid dendritic cells (pDCs) are the major producers of type I IFN in response to viral infection and have been shown to direct both innate and adaptive immune responses *in vitro*. However, *in vivo* evidence for their role in viral infection is lacking. We evaluated the contribution of pDCs to acute and chronic virus infection using the *feeble* mouse model of pDC functional deficiency. We have previously demonstrated that *feeble* mice have a defect in TLR ligand sensing. Although pDCs were found to influence early cytokine secretion, they were not required for control of viremia in the acute phase of the infection. However, T cell priming was deficient in the absence of functional pDCs and the virus-specific immune response was hampered. Ultimately, infection persisted in *feeble* mice. We conclude that pDCs are likely required for efficient T cell priming and subsequent viral clearance. Our data suggest that reduced pDC functionality may lead to chronic infection.

## Author Summary

The immune system consists of two arms aimed at fighting infection. Innate immunity represents the first barrier of defense to swiftly react – within minutes – following intrusion by a pathogen. Adaptive immunity is activated a few days later. Cross-talk between these two systems is critical but the means of communication are not yet fully understood. Plasmacytoid dendritic cells (pDCs) are innate immune cells best recognized for their ability to produce type I interferon (e.g. in response to viral infection.) Evidence for pDCs to modulate the adaptive system *in vivo* is only recent and still elusive. Using a newly described mouse model named *feeble* that is characterized by functional deficiency of pDCs, we analysed the role of *feeble* in the context of acute and chronic viral infection. We found that the *feeble* mutation affecting pDCs is dispensable for immunity during an acute infection. However our data show that *feeble* mice failed to control a chronic infection. This was likely due to a reduction in early cytokine secretion and improper activation of adaptive T cells, resulting in virus persistence. Therefore we propose that pDCs are critical for the resolution of chronic infection by linking both arms of immunity.

## Introduction

Plasmacytoid dendritic cells (pDCs) are a rare subset of DCs first appreciated in the blood and peripheral lymphoid tissues of humans and notable for producing extremely high amounts of type I interferon [1]. Thereafter, three studies reported the identification and characterization of the murine counterpart [2]–[4] which was paired with an increased interest and ability to understand pDC function *in vivo*. pDCs generate ∼1000× more type I interferon than any other cell type which accounts for the majority of circulating type I interferon during acute viral infections (reviewed in [5]). Therefore, it has been postulated that pDCs play a crucial role in disease and particularly viral infections [5]. Despite this tremendous biochemical capacity, a potent physiological role for pDCs *in vivo* has remained more difficult to identify. Part of this problem can be attributed to the lack of suitable tools to study pDC function *in vivo*. Several models have been recently created to address this issue including E2-2 deficient mice [6], BDCA-2-DTR [7] and Siglec-H-DTR [8] transgenic mice. In addition to the numerous activating functions demonstrated previously, some models have also demonstrated that pDCs can exert significant inhibitory function by controlling the homeostasis of regulatory CD4^+^ T cells [8]. Therefore, despite the classic immune stimulating functions first intimated for pDCs given their potent production of type I interferon, the ultimate role of pDCs in specific infections remains unclear.

Using a novel genetic screen we reported the utility of a mutant allele of *Slc15a4* (solute carrier family 15, member 4) named “*feeble*” to study pDC function *in vivo*. This ENU-induced single base pair transition resulted in pDCs unable to sense TLR ligands due to a defect in the SLC15A4 histidine transporter [9]. Importantly, TLR responses are specifically ablated in pDCs from *feeble* mice (a defect seen in the traditional *Slc15a4* knockout as well [10]), but intact in other cell types. Also, pDC number is maintained in mutants. Thus *feeble* mice provide a unique condition where pDCs are present but largely deficient for their function as producers of inflammatory cytokines [9]. This is in contrast to previous murine models involving deletion of pDCs, resulting in compensatory mechanisms within other immune effectors that may obscure the intrinsic role of pDCs [8].

To address these questions of pDC function *in vivo*, we used the natural mouse pathogen lymphocytic choriomeningitis virus (LCMV) in an acute and chronic infection model. *feeble* mutants displayed clearance kinetics similar to wild type mice after an acute LCMV Armstrong infection. In contrast, LCMV Clone 13 (Cl13) could not be contained by virus-specific T cells, thereby suggesting a role for pDCs in controlling persistent viral infections.

## Results/Discussion

### PDCs are dispensable for early virus control but critical for long-term clearance

**Figure 1:**
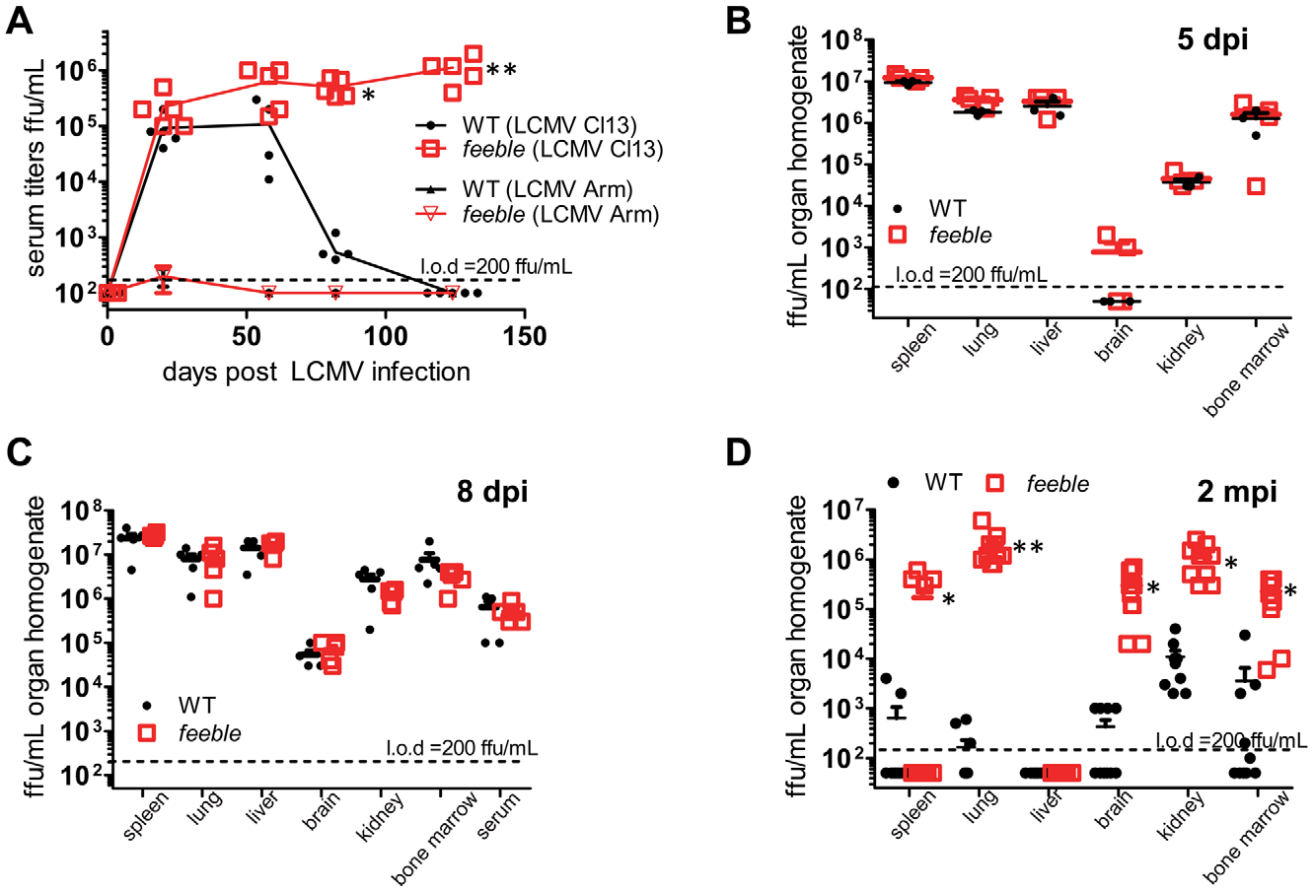
*Slc15a4* is required to control chronic viral infection. Serum and organs were collected at the indicated times post LCMV infection and infectious virus was titered. WT and *feeble* mice were infected i.v. with 2×10^6^ pfu of the acute LCMV Armstrong (Arm) strain or the persistent LCMV Clone 13 (Cl13) strain (A). Serum was then harvested at the indicated time points for >4 months to enumerate viremia. After Cl13 infection, organs were harvested from WT and *feeble* mice 5 dpi (B), 8 dpi (C), and 2 mpi (D), homogenized and titered for infectious virus. Individual replicates and means are shown. *Feeble*, *Slc15a4^feeble/feeble^* mice; l.o.d., limit of detection. Representative data of 2 independent experiments are shown; n> = 5 per group. Unless marked, p>0.05 between WT and *feeble* and not statistically significantly different.

In order to understand the functional role of pDCs *in vivo*, we challenged *feeble* mice with murine pathogens. We observed a striking deficit in the ability to control a persistent LCMV Cl13 infection without any significant change in the course of an acute infection from the parent LCMV Armstrong strain as measured by viremia over time (Figure 1A). To ensure whether this defect was specific to persistent (as opposed to acute infection), we harvested organs from LCMV Armstrong infected mice at several time points. LCMV Armstrong showed mild differences in infectious titer 5 days post infection (dpi) which were not present at 10 dpi (Figure S1) or anytime thereafter (for which we were unable to detect infectious virus), consistent with our serum titer data.

As pDCs are known to respond rapidly to an infectious challenge [5], [7], we reasoned that this defect in immunity may be due to a difference in Cl13 establishment during the initial phase of the infection. To address this possibility, we harvested organs from WT and *feeble* mice 5 and 8 days after Cl13 infection. However, we found no difference in viral titer or tropism at this stage of the infection (Figure 1BC). By 2 months post infection, and consistent with our serum titer data (Figure 1A), we observed that most organs known to harbor a persistent LCMV infection sustained very high levels of virus in *feeble* in contrast to WT mice (Figure 1D).

### PDC dysfunction results in reduced T cell priming

**Figure 2:**
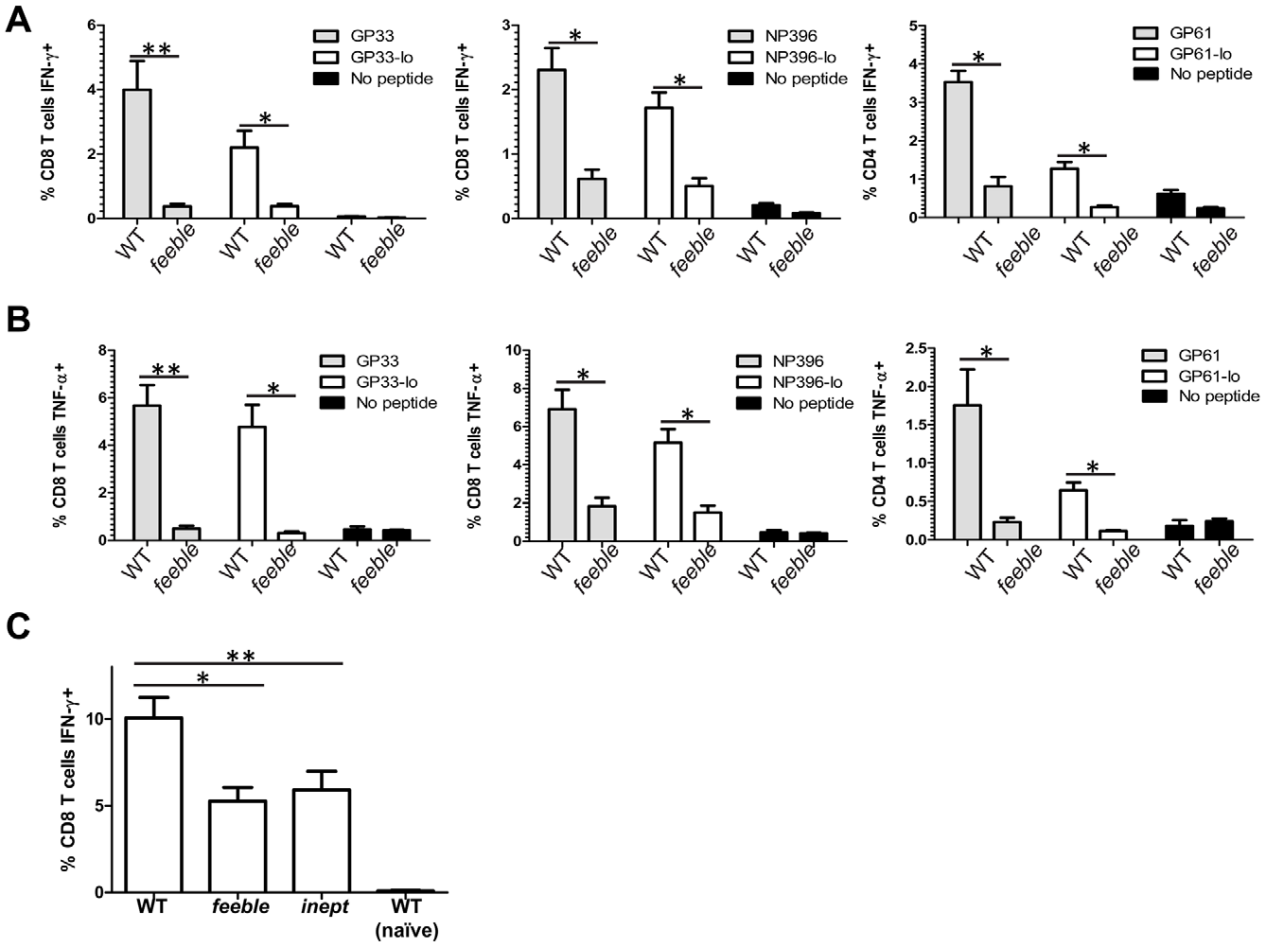
*Slc15a4* is required for antigen specific T cell responses *in vivo*. 8 days after LCMV Cl13 infection, splenocytes from WT and *feeble* mice were cultured *ex vivo* with the indicated peptides at 10^−7^ M, 10^−8^ M (“lo” concentration) or without peptide for 5 hours and then stained with antibodies to quantitate T cell antigen specific production of IFN-γ (A) and TNF-α (B). In (C) mice were primed with TAP1−/− 5E1 fibroblasts expressing the model antigen from Adeno E1B. Seven days after i.p. injection splenocytes from WT, *feeble*, and *inept* mice were cultured *ex vivo* with the immunodominant antigen for 5 hours. Then IFN-γ expression was quantitated in CD8^+^ T cells by flow cytometry. Mean and standard error of the mean are shown. *feeble*, *Slc15a4^feeble/feeble^* mice; *inept, IRF7^inept/inept^ mice*. Representative data of 2 independent experiments are shown. n≥4 per group of mice. Unless marked, p>0.05 between WT and *feeble* and not statistically significantly different.

Cognate T cell responses are critical in controlling persistent viral infections. This is well documented for LCMV Cl13 infection [11] but also for other, clinically relevant persistent infections such as HIV, HBV and HCV [12]. Therefore, we evaluated T cell function 8 days post LCMV Cl13 infection during the peak response. In comparison to WT infection, *feeble* displayed hypofunctional CD4^+^ and CD8^+^ T cell responses against immunodominant LCMV epitopes (Figure 2AB). Consistent with our findings in Figure 1, this defect was specific to a persistent infection and not observed during LCMV Armstrong infection (Figure S2.)

CD8^+^ T cell responses are instructed via both direct and cross-presentation. LCMV Cl13 like many other persistent pathogens directly inhibits APC function during infection [13]. In order to avoid viral immune evasion strategies, cross-presentation may be used by the host [14]. Thus, we reasoned that the *feeble* mutation may attenuate cross-priming. To address this we used a sterile model of inflammation depending on cross-presentation and type I IFN signaling [15]. Seven days after immunization, we observed a hypofunctional CD8^+^ T cell response in *feeble* as compared to WT animals, indicating a defect in priming (Figure 2C). As the magnitude of this defect was proportionally less than during Cl13virus infection (Figure 2C vs. Figure 2AB), we hypothesized that both pathways of antigen presentation require fully functional pDCs. Consistent with this idea, we observed that mice mutant in *IRF7 (IRF7^inept/inept^)*, which is required for the type I interferon response in pDCs, was similarly hypofunctional after sterile immunization (Figure 2C).

### Reduced T cell priming in feeble mice is due to a T cell extrinsic dysfunction

**Figure 3:**
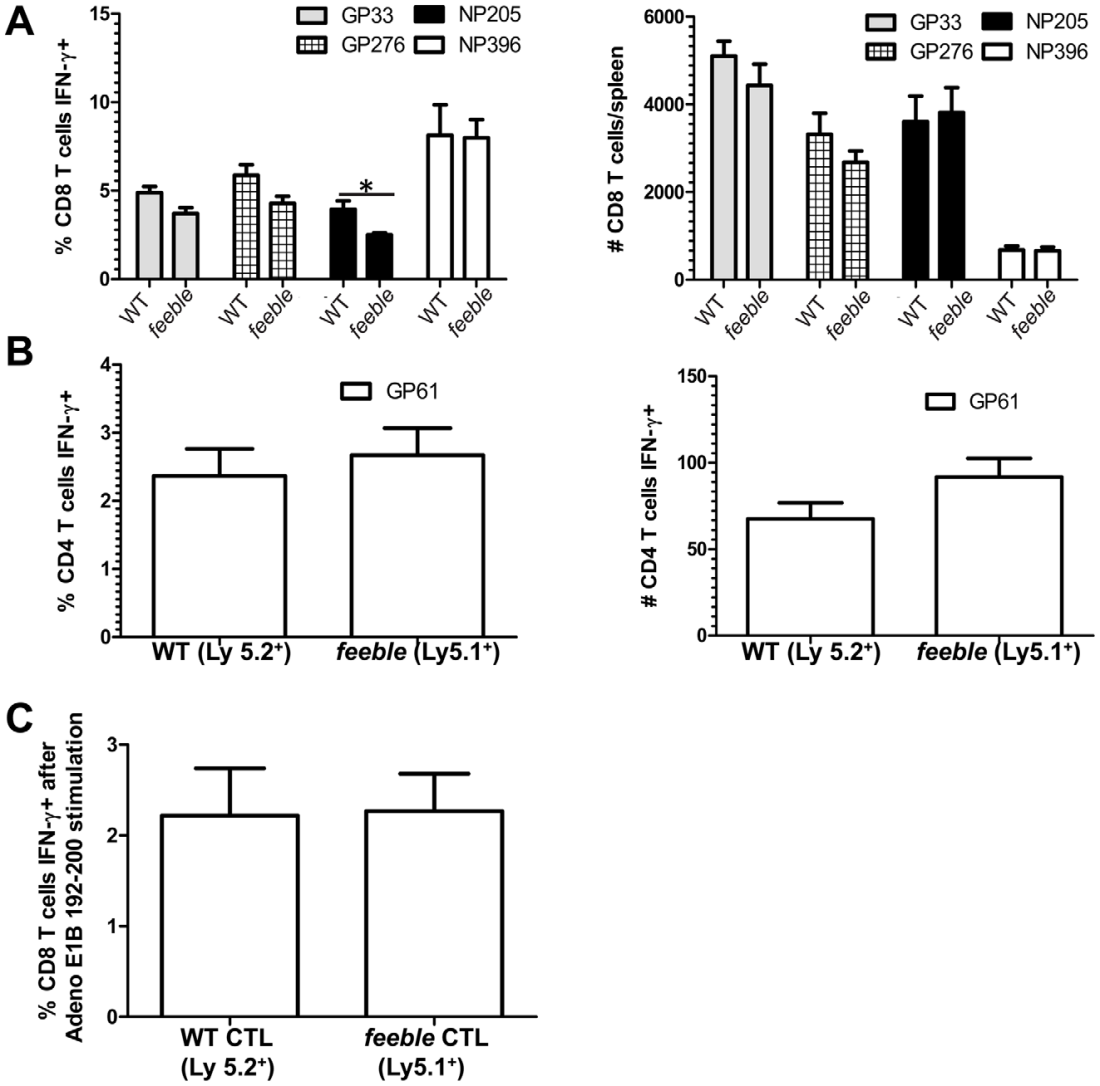
Defect in antigen specific *feeble* T cell responses is extrinsic. Equal numbers of bone marrow progenitors were harvested from WT and *feeble* mice and transferred into *feeble* recipients to generate mixed bone marrow chimeras. >8 weeks post irradiation, mixed bone marrow chimeras were challenged with LCMV Cl13. Eight days post infection splenocytes were harvested, counted, cultured with the indicated peptides for 5 hours, stained with antibodies, and analyzed by flow cytometry IFN-γ production in CD8^+^ (A) and CD4^+^ (B) antigen specific T cells. In (C) mice were primed with TAP1−/− 5E1 fibroblasts expressing the model antigen from Adeno E1B. Seven days after i.p. injection splenocytes from mixed bone marrow chimeras (prepared as above) were cultured *ex vivo* with the immunodominant antigen for 5 hours. Then IFN-γ expression was quantitated in CD8^+^ T cells by flow cytometry. Mean and standard error of the mean are shown. *Feeble*, *Slc15a4^feeble/feeble^* mice. n = 5 per group of mice. 1 of 2 similar experiments is shown. Unless marked, p>0.05 between WT and *feeble* and not statistically significantly different.

Previously, we reported that the *feeble* mutation acted in pDCs [9]. However, it was formally possible that this mutation in the histidine transporter SLC15A4 could compromise T cell function intrinsically. To test this hypothesis, we generated mixed bone marrow chimeras in which the irradiated recipient was reconstituted with an equal portion of WT and feeble bone marrow progenitor cells. In this setting we could determine whether there was a competitive advantage between WT and mutant cells. We did not detect a difference in the expansion nor functional activity of CD4^+^ and CD8^+^ T cells 8 days post LCMV Cl13 infection between WT and *feeble* cells (Figure 3AB). Similarly, when mixed bone marrow chimeras were challenged with an antigen that must be cross-presented, we did not observe any differences between WT and *feeble* (Figure 3C). Therefore, the defect in antigen specific *feeble* T cell responses was extrinsic to the T cells themselves.

To further address the location where the *feeble* mutation was acting, we made use of OT-I TCR transgenic mice bearing CD8^+^ T cells specific for the model antigen ovalbumin [16]. We generated OT-I; *feeble* double mutant mice and cultured naïve splenocytes *ex vivo* for 3 days in the presence of a greater than 1000-fold range of the cognate immunodominant epitope SIINFEKL. We chose to assess proliferation as it is known to be extremely sensitive to inhibition in CD8^+^ T cells [11]. We also focused our efforts on CD8^+^ T cells given their established importance in controlling persistent LCMV Cl13 infection [11] in contrast to other cell types (e.g. B cells, NK cells [17], [18]). No differences were observed between OT-I and OT-I; *feeble* double mutant mice with regards to proliferation (Figure S3). We concluded that the *feeble* mutation was not required for T cell function in this assay.

In addition, we considered whether the *feeble* mutation may affect other cell types intrinsically and therefore have a contributing role towards the observed *in vivo* phenotype (Figure 1, 2). Similarly to prior studies in which cDC function was intact in *feeble* mice [9], we did not observe a defect in NK cell cytotoxicity (Figure S4) or TLR responses of B cells and thioglycolate-elicited macrophages (personal observations).

Given the intact intrinsic lymphocyte function observed (Figures 3, S2, S3), we sought to evaluate other mechanisms which could compromise the adaptive T cell response *in vivo*.

### Diminished early inflammatory cytokine production in feeble mice following virus infection

**Figure 4:**
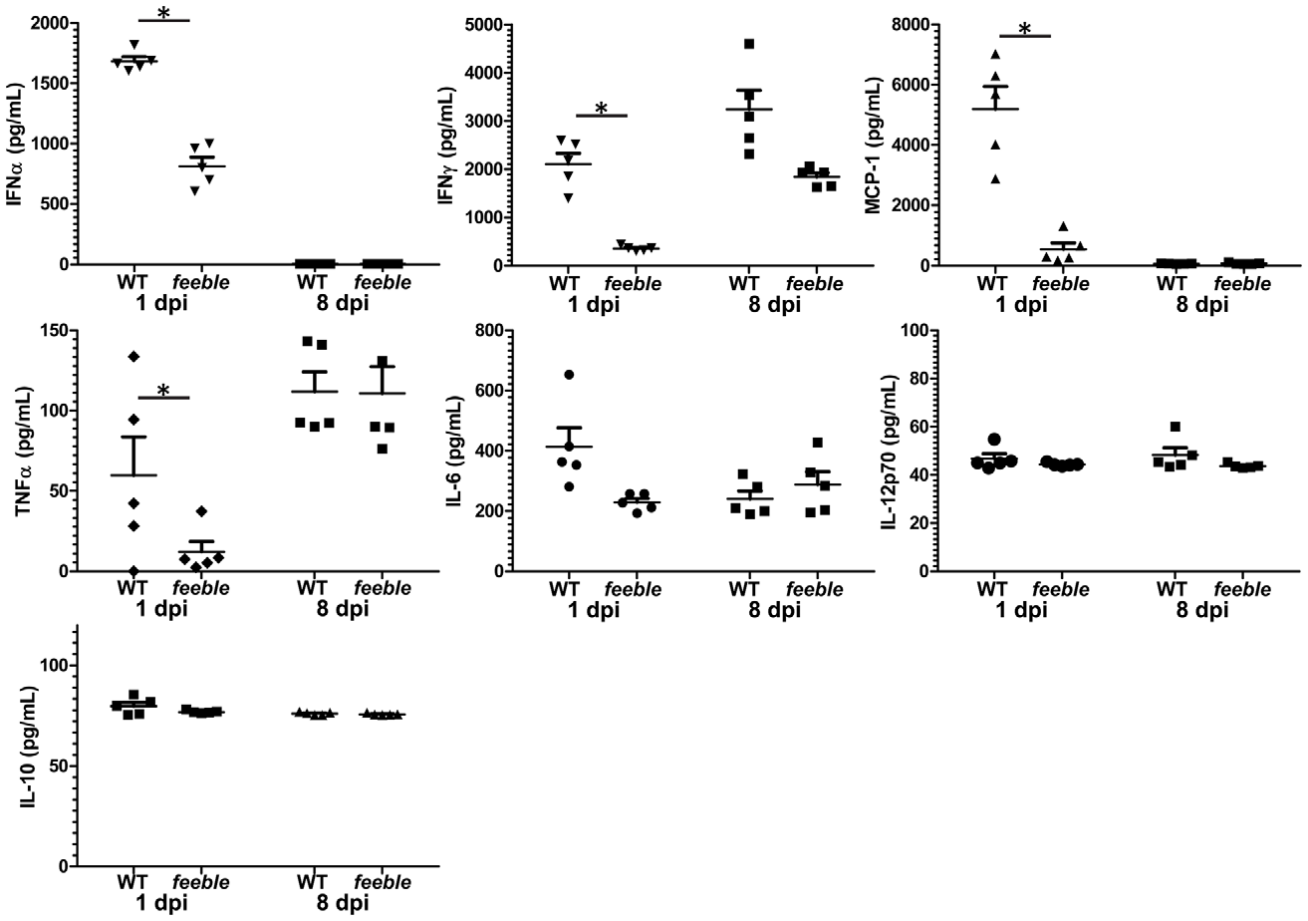
Slc15a4 is required for specific acute inflammatory cytokine production. One and 8 days post LCMV Cl13 infection serum was collected and the cytokines displayed were quantitated from WT and *feeble* mice. Individual replicates, mean and standard error of the mean are shown. Representative data of 2 independent experiments are shown; n = 5 per group of infected mice. Unless marked, p>0.05 between WT and *feeble* and not statistically significantly different.

pDC are best recognized for their abundant production of type I interferon and other cytokines [5]. Therefore we reasoned that a defect in these secreted molecules may contribute to virus susceptibility and a defect in antigen specific T cell activation (e.g. as shown via pDC derived type I interferon [19]–[23]). To address this possibility, we assessed cytokine/chemokine production 1 and 8 days post infection in the circulation as a potential contributing mechanism for our findings. We observed a reduction in several cytokines with an almost complete loss in the serum concentration of IFNγ and MCP-1 (Figure 4). These two factors are well known for their ability to both recruit and activate professional antigen presenting cells (pAPCs) and may be expressed by pDC in addition to other cell types. Therefore, we reasoned that a defect may lie in the recruitment and/or activation of pAPCs which could explain the *in vivo* specific defect in T cell activation in *feeble*. Splenocytes were harvested at 0, 1, 3 and 5 days post infection to quantitate both the numbers and activation of key immune effector cells early during LCMV Cl13 infection (Figure S5). We observed a mild difference in both the recruitment and activation of pAPCs as measured by the total number of viable cells and their functional markers MHC class I & class II in addition to the costimulatory molecules CD80 and CD86 on the three predominant pAPC (B cells, macrophages and dendritic cells) early during infection (Figure S5BC). Defects in both the total number and mean fluorescent intensity of functional markers were most pronounced 1 dpi although some deficits were observed 3 dpi. However, these differences were largely not statistically significant. Given the differences observed in cytokine/chemokine serum levels observed acutely post infection (Figure 4) in combination with a mild defect in the recruitment and activation of pAPC to the spleen, we hypothesize that these defects contribute in part to a more subtle and/or complex mechanism present early on in *feeble* resulting in attenuation of T cell responses, and that may involve other immune effectors. Additionally, our findings may underlie an underappreciated mechanism for pDC action.

### Prophylactic activation of pDCs prevents viral persistence

**Figure 5:**
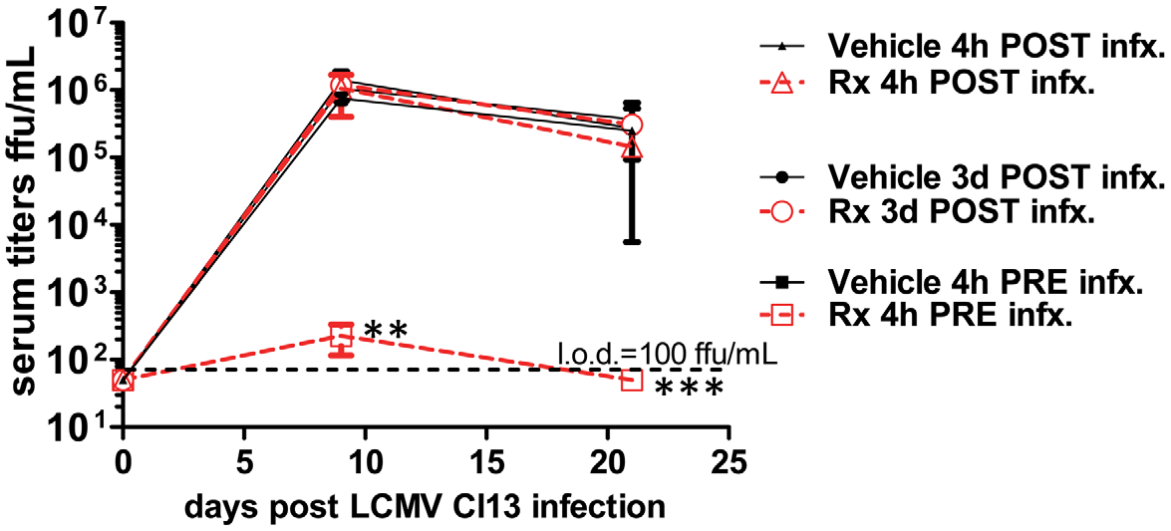
Prophylactic treatment with CpG-DOTAP prevents persistent viral infection. WT mice were treated with vehicle or i.v. 2 µg CpG-ODN in DOTAP (“Rx”) either 4 h prior, 4 h post or 3 days post infection with LCMV Cl13. Serum was collected at the indicated times post infection and titered for infectious virus. CpG-DOTAP: CpG-A plus DOTAP (Roche); l.o.d., limit of detection. Mean and standard error of the mean are shown for 5 mice per group of infected mice. 1 of 2 similar experiments is shown. Unless marked, p>0.05 between treatment groups and not statistically significantly different.

Given the dramatic requirement we observed for pDCs in controlling a persistent viral infection, we postulated that manipulation of their function may be of therapeutic benefit. To test this hypothesis, we used the TLR9 agonist type A cytosine guanine oligodeoxynucleotide (CpG-ODN). WT mice were given either vehicle or 2 ug CpG2216-ODN (a CpG motif specific for pDC [24]) in dinucleotide-1,2-dioleoyl-3-trimethylammonium-propane (DOTAP) 4 hours before and after LCMV Cl13 infection as well as 3 days post infection. We observed a window in which CpG-ODN was effective in preventing viremia when administered immediately prior to infection (Figure 5). Our findings demonstrate the limitations and a potential starting point for using pDC specific TLR agonists as an approach in the treatment of persistent viral infections. This may be due to the already chronic stimulation of pDCs under these conditions [5].

Nevertheless, our data also suggest a role for pDCs for the priming of sterile adaptive immunity, which may be translated into cancer therapy. Along this line, a recent study has shown that pDCs may be the key effectors in eliminating malignant melanoma with the clinically approved and prevalently used TLR7 agonist imiquimod [25].

Very recently, a manuscript has been published, showing that constitutive ablation of pDCs leads to virus persistence in mice [26]. While these results complement our data, they also illustrate differences. Contrary to pedigrees genetically lacking pDCs, *feeble* mutants have normal pDC numbers and are on a pure C57BL/6J background. Infection with 10^5^ pfu of LCMV Docile resulted in increased virus titers as early as 5 days post infection in pDC lacking mice [26], while we observed prominently higher viremia in *feeble* mutants only 2 months after challenge with 2×10^6^ pfu LCMV Clone 13 (Figure 1D), and not during the early stage of the infection. Together with the reduction in priming of *feeble* CD8^+^ T cell upon sterile immunization (Figure 2C) our data demonstrate that the impaired T cell function in absence of functional pDCs is not secondary to failure in early virus control, as it may occur for viral strains with high replication kinetics [27]. We also show that the *feeble* mutation negatively impacts on the production of several pro-inflammatory cytokines (Figure 4), yet without severely affecting the recruitment and activation of pAPCs (Figure S5).

Furthermore, another group used the transgenic BDCA2-DTR mouse [7] to deplete pDC during the revision of this manuscript [28]. Interestingly, this study using LCMV Clone13 did not see a difference in viremia upon depletion of pDC with diphtheria toxin. As this model of pDC depletion results in ∼90% loss of pDCs, we suspect that this discrepancy in findings may be due to residual function of the remaining pDC in the BDCA2-DTR mouse model.

Regarding the timing of effects in our model, we note that significant defects in CD4+ and CD8+ T cell function are observed 8 days post infection. At this time, the amount of infectious virus in several organs and the blood stream of *feeble* mice are very similar to WT animals. However, subsequent to this timepoint (8 dpi) we observe higher systemic viral loads in *feeble* versus WT mice. As pDC activity is seen early by ourselves (Figure 5) and others (reviewed in [5]) one may expect even higher viral loads during the first week of infection in *feeble* mice, which we do not observe. This may reflect a physiological “ceiling” of maximum viral load allowed by the mouse at this time. For instance, when analyzing mice deficient in type I, type II or both interferon receptors as compared to WT mice [29], splenic viral loads were very similar between all four strains of mice during the first week of infection. Differences between these strains were only noticed subsequent to this time despite our knowledge of the seminal role of interferons in the control of viral infection. Likewise, in our studies we observe very early defects in cytokine production (1 dpi) and early (8 dpi) defects in T cell function. However, the differences in viral loads are not observed until after 8 dpi. CD4^+^ and CD8^+^ T cells are required for control of persistent LCMV infection and adoptive transfer of these cells is sufficient to eliminate a persistent LCMV infection [30], [31]. Therefore, we suspect that like interferon receptor deficient animals a maximum level of viremia is established during the first 8 days of infection which is ultimately cleared by the combined effort of CD4^+^ and CD8^+^ T cells in WT mice. However, *feeble* mice lack effective CD4^+^ and CD8^+^ T cell responses (although the total # of splenocytes at this time is unchanged, data not shown) and are unable to clear systemic infection during our studies. We suspect that the defect in T cell function accounts for the inability of *feeble* mice to control persistent viral infection.

Interestingly, the gene mutated in *feeble* mice, *slc15a4*, has been implicated in NOD1 signaling *in vivo* [10]. Therefore, it is possible that incomplete NOD1 signaling may contribute to defects in controlling persistent viral infection in the presence of mutant *slc15a4*. However, we believe this to be less likely than a role for pDC function given the body of literature supporting NOD1 (a receptor for peptidoglycan derivatives) function in bacterial infections [32] versus the role of pDC in viral infections [5].

PDCs have also been implicated in the control of persistent infections in humans. This is largely due to their potential function for type I interferon production which directly augments T cell function [19]–[23] and the observation that persistent infectious viruses (e.g. HIV, HBV, HCV) actively suppress pDC function [5], [33], [34]. Moreover, it is known that patients with higher HIV viral loads and lower CD4^+^ T cell counts have a deficit in pDC numbers which are not recovered with highly active anti-retroviral therapy (HAART) (reviewed in [5]). Therefore, our work gives a functional framework to better understand these observations. An important extension of this work will be to address whether a subset of HIV, HBV or HCV patients who are more susceptible to disease progression have a functional compromise in pDC function encoded genetically or otherwise that can be modulated therapeutically. Our studies may provide a foundation to address these and other clinically relevant questions pertaining to pDCs and human disease.

## Materials and Methods

### Ethics statement

This study was carried out in strict accordance with the recommendations in the Guide for the Care and Use of Laboratory Animals of the National Institutes of Health. The protocols were approved by the Institutional Animal Care and Use Committee of The Scripps Research Institute (Protocol#:09-0098) as well as the Institutional Animal Care and Use Committee of Case Western Reserve University (Protocol#:2011-0200).

### Mice

C57BL/6J mice were bred locally or ordered from Jackson Laboratories. *Slc15a4^feeble/feeble^* (MGI: 4835997) and *Irf7^inept/inept^* (MGI: 4442855) mutants have been described previously [9]. OT-I mice have been described previously [16]. Compound mutants were generated by intercrossing F1 progeny.

### Viruses, focus-forming unit (FFU) assay

The Armstrong strain and Clone 13 variant of LCMV was injected intravenously at a dose of 2×10^6^ PFU per mouse. Viral titers were determined from serial dilutions of serum and organ homogenate by a focus-forming assay on VeroE6 cells as described previously [35].

### Antibodies, intracellular cytokine staining and *ex vivo* restimulation of T cells

The following antibodies were used for flow cytometry, to stain splenocytes: CD3ε (145-2C11, eBioscience), CD4 (L3T4, eBioscience), CD8α (53-6.7, eBioscience), CD19 (MB19-1, eBioscience), F4/80 (BM8, Biolegend), Mac-1 (M1/70, Biolegend), CD80 (B7-1, Biolegend), CD86 (GL1, Biolegend), MHC Class I (28-14-8, Biolegend), MHC Class II (M5/114.15.2, Biolegend), IFNγ (XMG1.2, Biolegend), TNFα (MP6-XT22, Biolegend). Cell populations were defined as follows: T cell populations, CD3ε^+^ and either CD4^+^ or CD8^+^; B cells, CD19^+^, CD3^−^,CD11c^−^; macrophages, F4/80^hi^, Mac-1^hi^; dendritic cells CD11c^hi^. Specific T-cell responses were determined *ex vivo* by intracellular IFNγ and TNFα staining after a 5 hour stimulation with 10^−7^ M peptide in the presence of brefeldin A (BD Biosciences) as described before [35] unless otherwise indicated.

### Antigen challenge with 5E1 cells

*Tap1^−/−^* 5E1 mouse embryonic fibroblast cells were used to access for a *feeble* dependent role in cross presentation. 10^7^ 5E1 cells were given i.p. per mouse. These cells express the Adeno E1B 192-200 immunodominant Db-restricted peptide [15]. Cells were irradiated with 3000 rad prior to injection. Seven days after injection, splenocytes were harvested and cultured *ex vivo* with 10^−7^ M VNIRNCCYI for 5 hours. Then CD8^+^ T cell intracellular cytokine production was quantitated by flow cytometry.

### Serum cytokine detection

Blood samples were taken from the retro-orbital plexus of mice. Cytokines (IFNγ, MCP-1, TNFα, IL-6, IL-12p70 and IL-10) were enumerated with the BD Mouse Inflammatory Cytometric Bead Array per manufacturer's instructions. IFNα was enumerated by VeriKine Mouse Interferon-Alpha ELISA Kit per manufacturer's instructions.

### Ovalbumin induced expansion of CD8^+^ T cells in OT-1

OT-1 transgenic mice [16] were bred onto the *feeble* allele to produce double mutants. Splenocytes were harvested from OT-1; *feeble* and OT-1 mice and incubated *ex vivo* for 3 days with the immunodominant CD8^+^ ovalbumin epitope SIINFEKL under a range of concentrations. CD8^+^ transgenic T cell proliferation was quantitated with carboxyfluorescein succinimidyl ester (CFSE) staining detected by flow cytometry and analyzed with FlowJo software.

### Mixed bone marrow chimera production

For bone marrow transplantation, bone marrow cells were extracted from femurs and tibias and were placed in PBS, 0.1% BSA (vol/vol). Equal numbers of bone marrow cells from congenic WT (C57BL/6.SJL) (PtprcaPep3b; Ly5.1^+^) and homozygous *feeble* mice (Ly5.2^+^) were injected intravenously into the lateral tail veins of recipient *feeble* mice irradiated (1000 rad) 24 h earlier.

### In vivo NK cell cytotoxicity assay

A total of 10^7^ control C57BL/6J splenocytes or TAP1-deficient splenocytes were resuspended in 1 mL PBS and labeled with low (0.5 µM) and high (5 µM) concentrations of carboxyfluorescein diacetate succinimidyl ester (CFSE) (Sigma-Aldrich), respectively, at room temperature for 10 min. The labeling was stopped by addition of cold FCS. Cells were washed twice, counted, and resuspended at a concentration of 5×10^7^ cells/mL. The two populations were mixed at a 1∶1 ratio and injected i.v. into recipient mice. Recipients were bled 24 hours later, and PBMCs were analyzed for CFSE staining by flow cytometry.

### Statistics

The statistical significance of differences was determined by unpaired Student's two-tailed t-test. Differences with a P value of less than 0.05 were considered statistically significant. For all figures: *P<0.05; **P≤0.01; ***P≤0.001. Unless marked, p>0.05 between WT and *feeble* and data groups were not statistically significantly different. Error bars show standard error of the mean (SEM).

## Supporting Information

Figure S1 ***Slc15a4*** **is not required to control acute viral infection.** Serum and organs were collected at the indicated times post LCMV Armstrong infection and infectious virus was titered. WT and *feeble* mice infected i.v. with 2×10^6^ pfu of the acute LCMV Armstrong strain. Animals were then sacrificed and the indicated tissue was isolated for viral titers at 5 and 10 dpi. Representative data of 2 independent experiments are shown; n> = 3 per group. Unless marked, p>0.05 between WT and *feeble* and not statistically significantly different.
(TIF)

Figure S2 ***Slc15a4*** **is not required for antigen specific T cell responses against an acute LCMV Armstrong infection** ***in vivo*** **.** 8 days after LCMV Cl13 infection, splenocytes from WT and *feeble* mice were cultured *ex vivo* with the indicated peptides or without peptide for 5 hours and then stained with antibodies to quantitate T cell antigen specific production of IFN-γ as well as double production of IFN-γ, TNF-α (CD8 T cells) and IFN- γ, IL-2 (CD4 T cells), as labeled. Mean and standard error of the mean are shown, N = 3. *feeble*, *Slc15a4^feeble/feeble^* mice. Representative data of 2 independent experiments are shown. There was no statistical difference between WT and *feeble*.
(TIF)

Figure S3 ***feeble*** **CD8 T cells and antigen presenting cells do not display defects** ***ex vivo*** **.** Spleens were harvested from naïve adult OT-I and OT-I; *feeble* mice. Whole splenocytes were then cultured for 3 days with CFSE over a range of specific peptide concentrations as indicated. Antigen induced cell proliferation was enumerated by flow cytometry. One of 2 representative experiments is shown. *feeble*, *Slc15a4^feeble/feeble^* mice. OT-I, ovalbumin-specific TCR transgenic line specific for the CD8^+^ T cell immunodominant epitope SIINFEKL.
(TIF)

Figure S4 **Intact intrinsic function of homozygous** ***Slc15a4^feeble/feeble^*** **NK cells.** Percentage of CFSE-labeled Tap1^−/−^ cells remaining in the blood of Tap1^−/−^, C57BL/6J, and *feeble* mice 24 h after injection of a 1∶1 mixture of CFSE-labeled Tap1^−/−^ and WT splenocytes was measured by flow cytometry. 1 of 2 independent experiments is shown with the same results. There was no statistical difference between WT and *feeble*.
(TIF)

Figure S5 **Immune cell recruitment and activation early during splenic infection.** Splenocytes were harvested from WT and *feeble* mice 0, 1, 3 and 5 days post infection (A–D, respectively) with LCMV Clone 13. Cells were counted and stained with antibodies to surface markers to distinguish B cell, T cell, dendritic cell (DC) and macrophage cell numbers. Cells were also stained with antibodies to MHC Class I, Class II, CD80 and CD86 to assess the activation status of B cells, macrophages and DCs. Individual replicates, mean and standard error of the mean (where indicated for total viable cells) are shown. n = 5 per group of infected mice. 1 of 2 similar experiments is shown. Unless marked, p>0.05 between WT and *feeble* and not statistically significantly different.
(PDF)
