# Supplementary figures and images for: Slc15a4, a Gene Required for pDC Sensing of TLR Ligands, Is Required to Control Persistent Viral Infection
Source: PLoS Pathog. 2012 Sep 13;8(9):e1002915. doi: 10.1371/journal.ppat.1002915 (PMC3441671; doi:10.1371/journal.ppat.1002915)

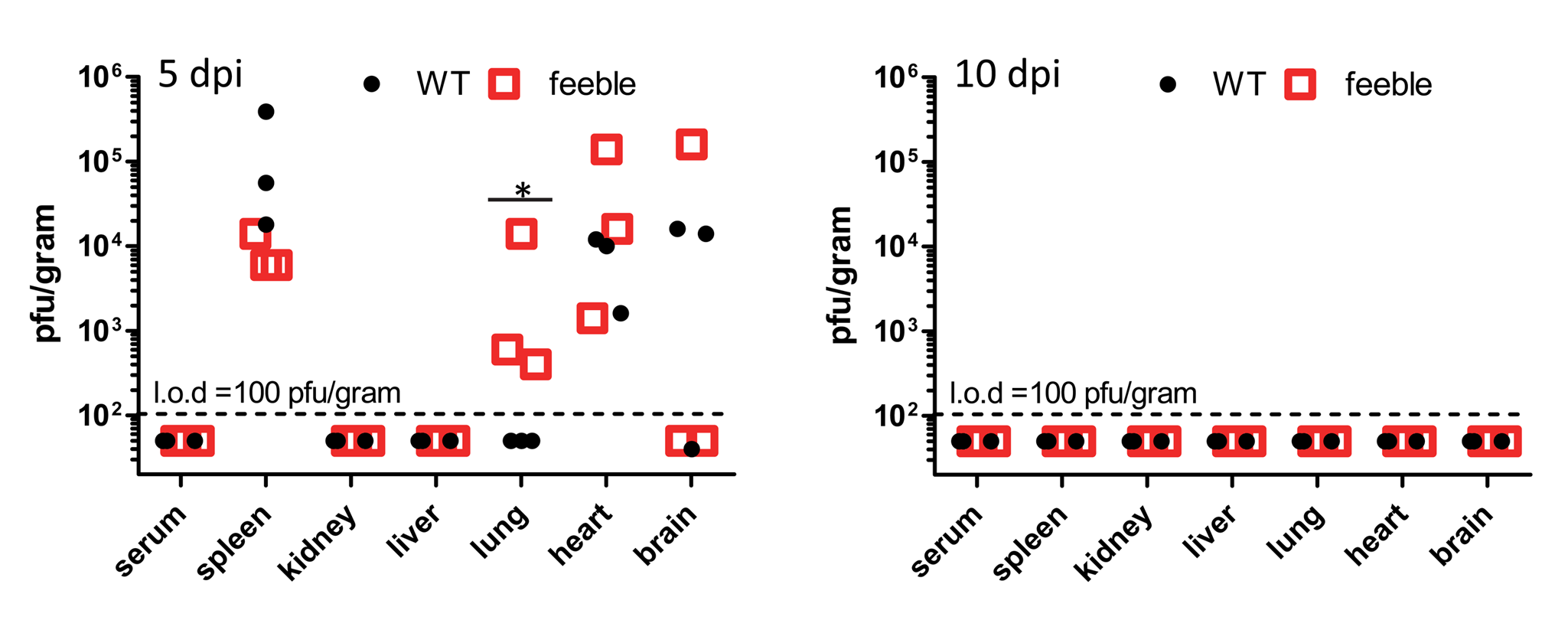

Supplement: Figure S1 — Slc15a4 is not required to control acute viral infection. Serum and organs were collected at the indicated times post LCMV Armstrong infection and infectious virus was titered. WT and feeble mice infected i.v. with 2×10^6^ pfu of the acute LCMV Armstrong strain. Animals were then sacrificed and the indicated tissue was isolated for viral titers at 5 and 10 dpi. Representative data of 2 independent experiments are shown; n> = 3 per group. Unless marked, p>0.05 between WT and feeble and not statistically significantly different. (TIF) [file ppat.1002915.s001.tif]

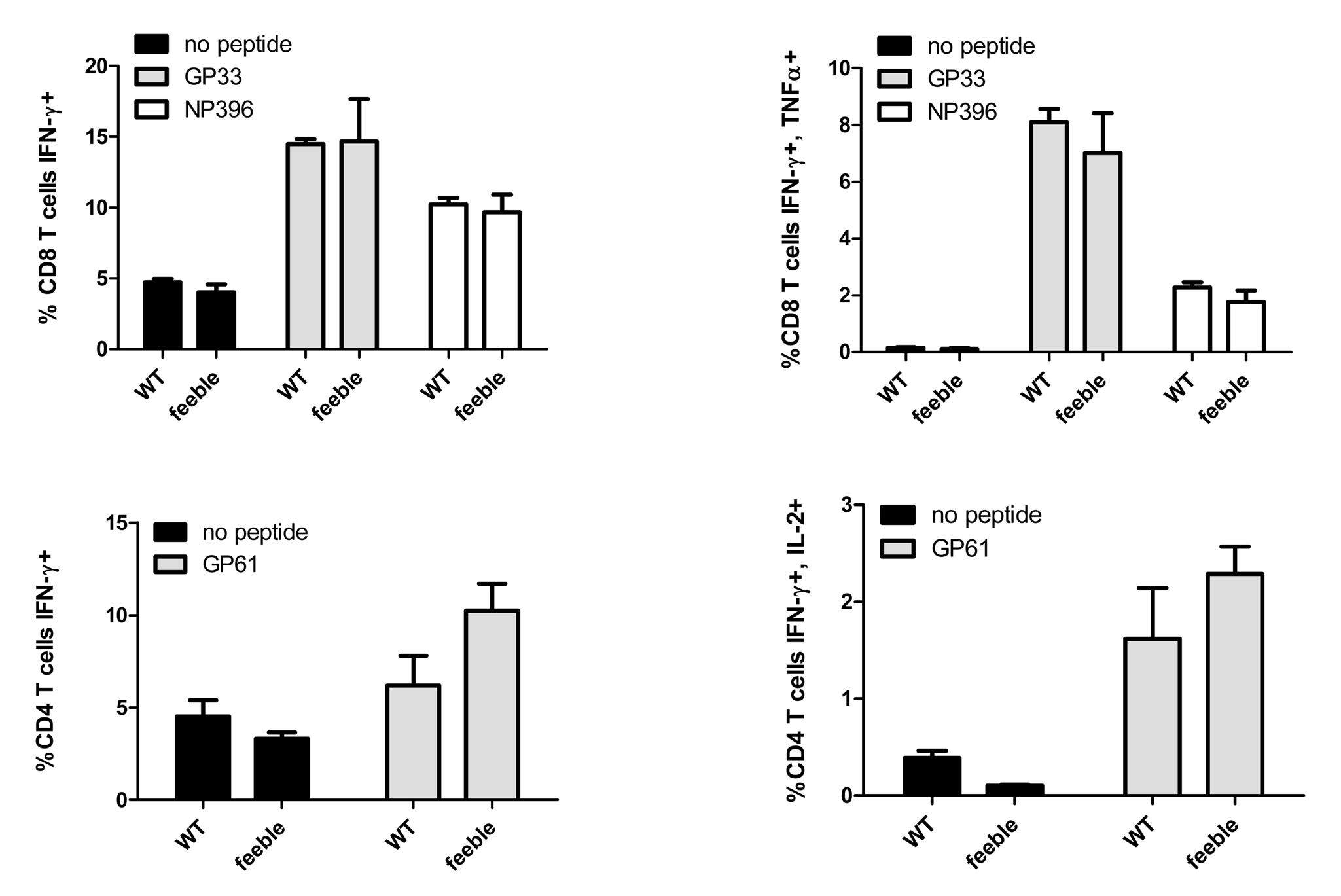

Supplement: Figure S2 — Slc15a4 is not required for antigen specific T cell responses against an acute LCMV Armstrong infection in vivo . 8 days after LCMV Cl13 infection, splenocytes from WT and feeble mice were cultured ex vivo with the indicated peptides or without peptide for 5 hours and then stained with antibodies to quantitate T cell antigen specific production of IFN-γ as well as double production of IFN-γ, TNF-α (CD8 T cells) and IFN- γ, IL-2 (CD4 T cells), as labeled. Mean and standard error of the mean are shown, N = 3. feeble, Slc15a4feeble/feeble mice. Representative data of 2 independent experiments are shown. There was no statistical difference between WT and feeble. (TIF) [file ppat.1002915.s002.tif]

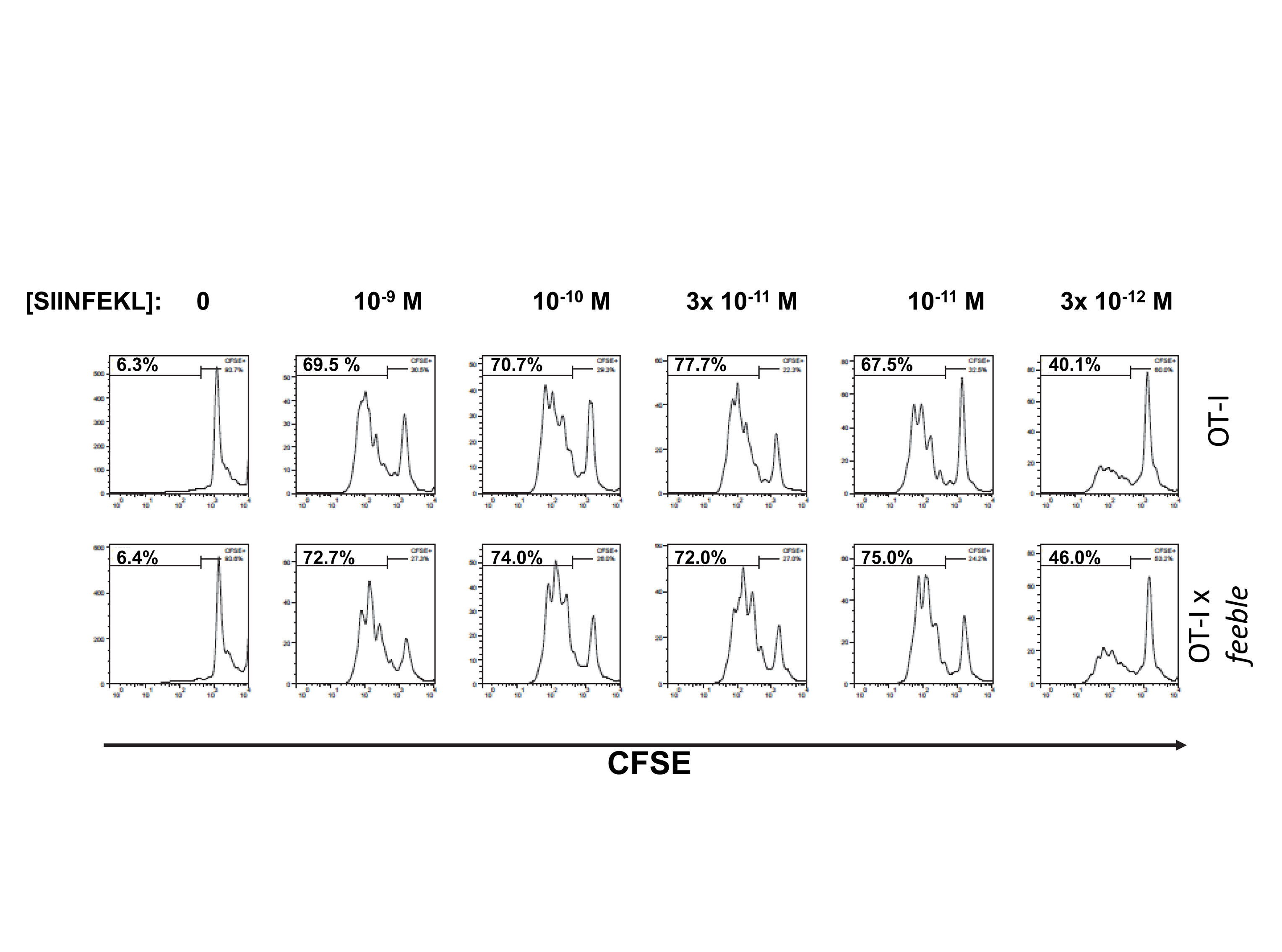

Supplement: Figure S3 — feeble CD8 T cells and antigen presenting cells do not display defects ex vivo . Spleens were harvested from naïve adult OT-I and OT-I; feeble mice. Whole splenocytes were then cultured for 3 days with CFSE over a range of specific peptide concentrations as indicated. Antigen induced cell proliferation was enumerated by flow cytometry. One of 2 representative experiments is shown. feeble, Slc15a4feeble/feeble mice. OT-I, ovalbumin-specific TCR transgenic line specific for the CD8+ T cell immunodominant epitope SIINFEKL. (TIF) [file ppat.1002915.s003.tif]

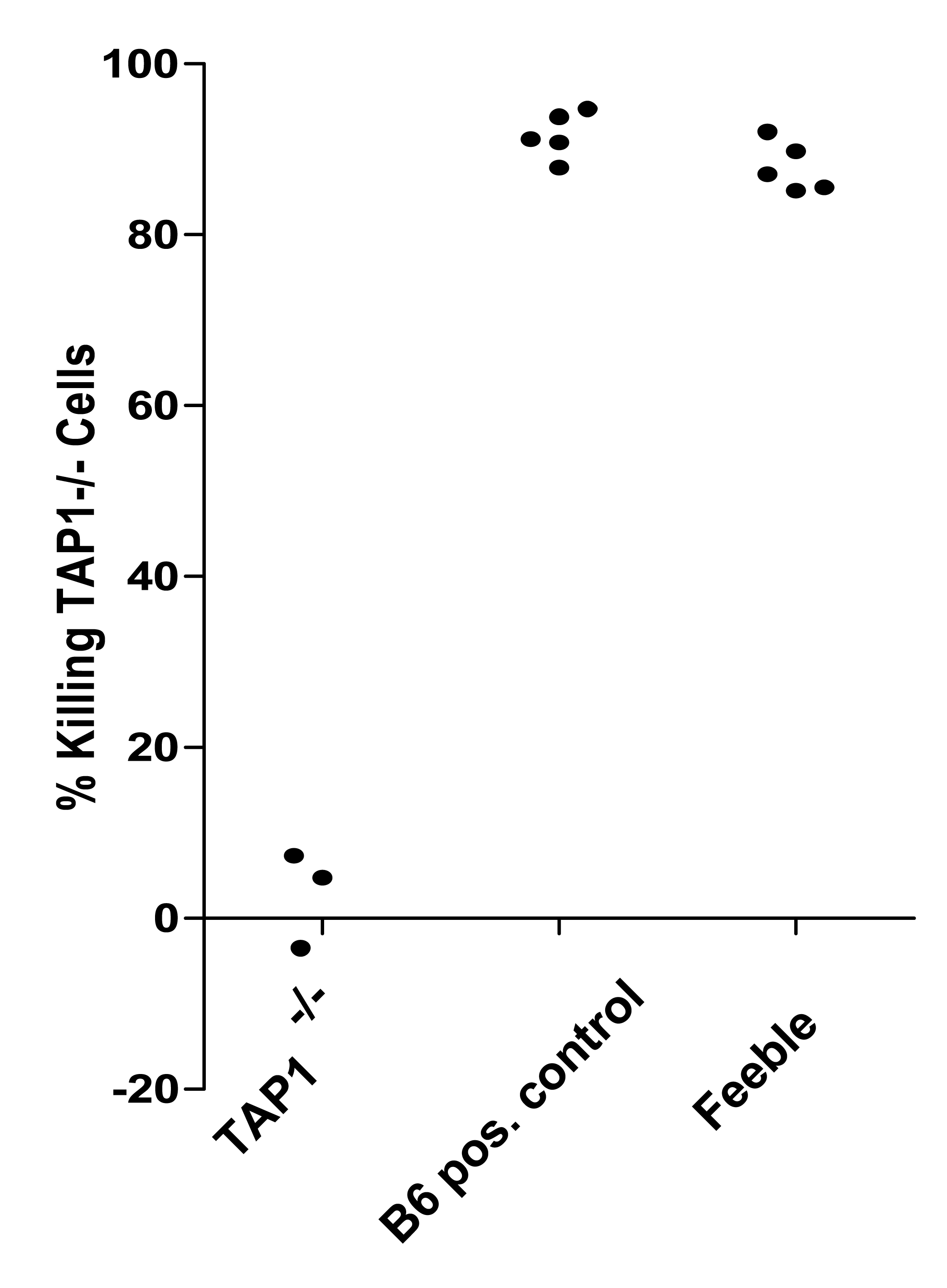

Supplement: Figure S4 — Intact intrinsic function of homozygous Slc15a4feeble/feeble NK cells. Percentage of CFSE-labeled Tap1−/− cells remaining in the blood of Tap1−/−, C57BL/6J, and feeble mice 24 h after injection of a 1∶1 mixture of CFSE-labeled Tap1−/− and WT splenocytes was measured by flow cytometry. 1 of 2 independent experiments is shown with the same results. There was no statistical difference between WT and feeble. (TIF) [file ppat.1002915.s004.tif]

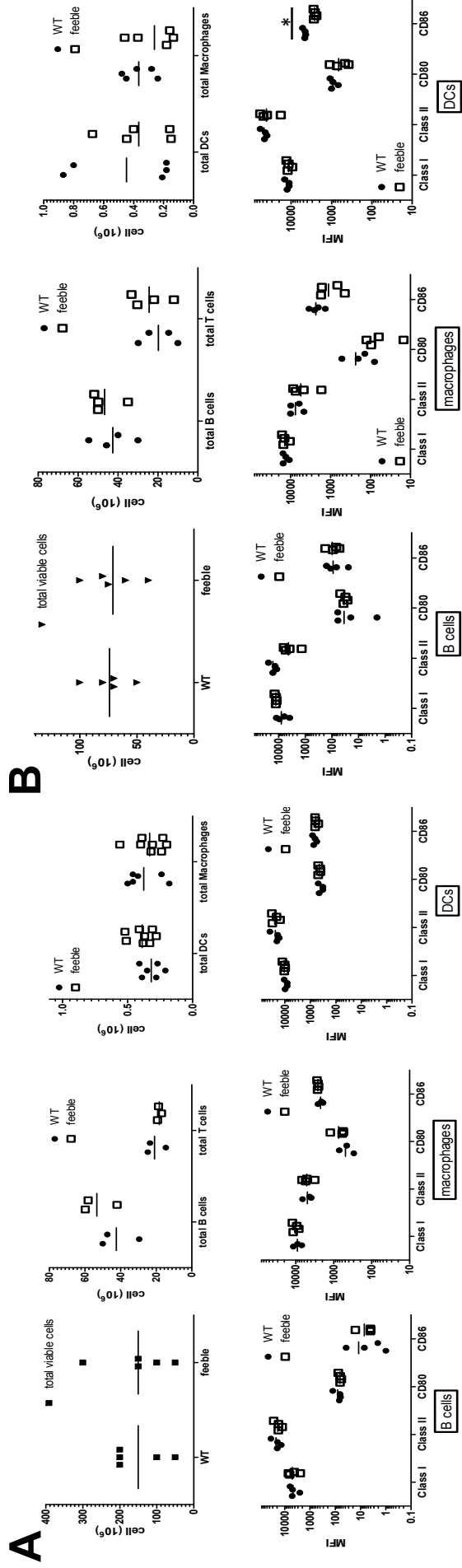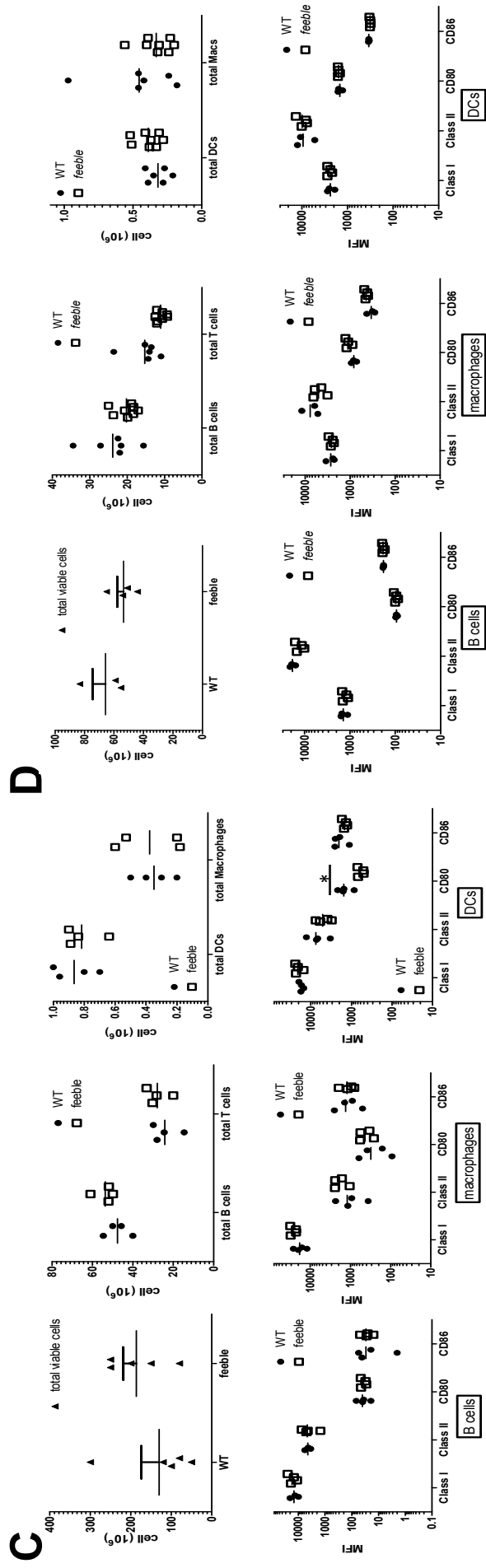

Supplement: Figure S5 — Immune cell recruitment and activation early during splenic infection. Splenocytes were harvested from WT and feeble mice 0, 1, 3 and 5 days post infection (A–D, respectively) with LCMV Clone 13. Cells were counted and stained with antibodies to surface markers to distinguish B cell, T cell, dendritic cell (DC) and macrophage cell numbers. Cells were also stained with antibodies to MHC Class I, Class II, CD80 and CD86 to assess the activation status of B cells, macrophages and DCs. Individual replicates, mean and standard error of the mean (where indicated for total viable cells) are shown. n = 5 per group of infected mice. 1 of 2 similar experiments is shown. Unless marked, p>0.05 between WT and feeble and not statistically significantly different. (PDF) [file ppat.1002915.s005.pdf]
